# Challenges in describing the conformation and dynamics of proteins with ambiguous behavior

**DOI:** 10.3389/fmolb.2022.959956

**Published:** 2022-08-03

**Authors:** Joel Roca-Martinez, Tamas Lazar, Jose Gavalda-Garcia, David Bickel, Rita Pancsa, Bhawna Dixit, Konstantina Tzavella, Pathmanaban Ramasamy, Maite Sanchez-Fornaris, Isel Grau, Wim F. Vranken

**Affiliations:** ^1^ Structural Biology Brussels, Vrije Universiteit Brussel, Brussels, Belgium; ^2^ Interuniversity Institute of Bioinformatics in Brussels, VUB/ULB, Brussels, Belgium; ^3^ VIB-VUB Center for Structural Biology, Brussels, Belgium; ^4^ Research Centre for Natural Sciences, Institute of Enzymology, Budapest, Hungary; ^5^ IBiTech-Biommeda, Universiteit Gent, Gent, Belgium; ^6^ VIB-UGent Center for Medical Biotechnology, Universiteit Gent, Gent, Belgium; ^7^ Department of Computer Sciences, University of Camagüey, Camagüey, Cuba; ^8^ Information Systems, Eindhoven University of Technology, Eindhoven, Netherlands

**Keywords:** protein dynamics and conformation, sequence-based prediction, biophysical characteristics, post-translational modification (PTM), deleterious mutation, folding-upon-binding, fold switching

## Abstract

Traditionally, our understanding of how proteins operate and how evolution shapes them is based on two main data sources: the overall protein fold and the protein amino acid sequence. However, a significant part of the proteome shows highly dynamic and/or structurally ambiguous behavior, which cannot be correctly represented by the traditional fixed set of static coordinates. Representing such protein behaviors remains challenging and necessarily involves a complex interpretation of conformational states, including probabilistic descriptions. Relating protein dynamics and multiple conformations to their function as well as their physiological context (e.g., post-translational modifications and subcellular localization), therefore, remains elusive for much of the proteome, with studies to investigate the effect of protein dynamics relying heavily on computational models. We here investigate the possibility of delineating three classes of protein conformational behavior: order, disorder, and ambiguity. These definitions are explored based on three different datasets, using interpretable machine learning from a set of features, from AlphaFold2 to sequence-based predictions, to understand the overlap and differences between these datasets. This forms the basis for a discussion on the current limitations in describing the behavior of dynamic and ambiguous proteins.

## 1 Introduction

The importance of protein dynamics for their (mis-)folding (Daggett and Fersht, 2003; Dobson, 2003) and functionality (Karplus and Kuriyan, 2005; Glazer, Radmer, and Altman, 2009) has been long recognized but has been overshadowed by the need to first understand how most proteins fold into well-defined three-dimensional structures (unique conformations) (Hunkapiller, Strickler, and Wilson, 1984; Berman et al., 2007). The recent impressive performance of AlphaFold2 (Jumper et al., 2021) in predicting such unique protein folds from i) protein sequence and evolutionary information curated by UniProt (The UniProt Consortium, 2021) and ii) the carefully assembled protein structure information from the Protein Data Bank over many decades (Berman et al., 2007) indicates that this problem is now largely solved. This also implies that experimental and computational approaches for proteins will now have to necessarily focus beyond their fold, specifically on understanding more about how proteins interact, which alternative conformations they might adopt, and how they move between these conformations. Indeed, many proteins show ambiguous conformational behavior, either in specific regions within folded domains [e.g., loops such as CDRs in antibodies (Armstrong, Piepenbrink, and Baker, 2008) or extracellular loops in GPCRs (Hilger, Masureel, and Kobilka, 2018)], in regions connecting folded domains [e.g., PEVK domain of titin (Hsin et al., 2011)], or the full protein in the case of intrinsically disordered proteins [e.g., Phd antitoxin from Bacteriophage P1 (De Gieter et al., 2014)]. This behavior does not have hard boundaries. For example, systematic studies on ambiguous/disordered proteins have already proved that missing residues in crystal structures do not always correlate with protein disorder. In fact, sometimes they are predicted as highly ordered (Gall et al., 2007). Similarly, residues that are present or missing for the same protein in different X-ray structures are rarely statically disordered and show a partial or conditional disorder under different experimental conditions (DeForte and Uversky, 2016). This different degree of disorder was previously described and categorized into foldable, non-foldable, or semi-foldable regions, where some protein regions undergo a structural rearrangement at a certain point in time, either spontaneously or induced (e.g., after binding with another molecule) (Uversky, 2013). These conformational changes often condition the functions that the proteins perform and break with the classical protein structure-function paradigm (Uversky, 2019), supporting the prevalence and importance of the ambiguous behavior that we are addressing. The move from the traditional paradigm, with the sequence encoding for a single static structure, toward a dynamic paradigm, where the sequence encodes for different possible behaviors, also implies the necessity to approach proteins from a probabilistic viewpoint. This is a reasonable assumption, especially when considering that billions of copies of the same protein exist in cells at thermodynamically high temperatures; all these proteins will have different interactions and (locally) different conformations at any given time point and might have (different) post-translational modifications (Vu, Gevaert and De Smet, 2018). Such a proteomics-based probabilistic *in vivo* view of proteins is in stark contrast to the reductionist and static single-protein view in the traditional paradigm.

There have nevertheless been significant efforts in the experimental investigation of the conformational ambiguity and heterogeneity of protein structures and structural ensembles by various techniques: nuclear magnetic resonance (NMR), circular dichroism (CD) and electron paramagnetic resonance (EPR) spectroscopy, small-angle X-ray and neutron scattering (SAXS/SANS), Förster resonance energy transfer (FRET) measurements, electrospray ionization–ion mobility mass spectrometry (ESI/IM-MS), and hybrid approaches that integrate more than one of the above-mentioned techniques (Dobson, 2019). Although X-ray crystallography and cryo-electron microscopy may both be able to trap more than one protein conformer of globular proteins, solution techniques are undoubtedly preferred for uncovering the dynamics of flexible proteins, with NMR being the approach that initially highlighted these features in proteins using different types of measurements (chemical shifts, R1, R2, J-couplings, NOEs, and RDCs). Lately, there have also been efforts dedicated to studying the dynamics of flexible and intrinsically disordered proteins (IDPs) in the cellular context using *in-cell* NMR and EPR spectroscopy, as a protein’s conformational behavior may differ from what is observed in isolation in the test tube (Gerez, Prymaczok, and Riek, 2020; Bonucci et al., 2021). However, due to various experimental challenges, these methods have not become widely used in the community of structural biology. Valid future alternatives for both single proteins (folding) and in-cell determination of protein states might come from mass spectrometry-based methods such as cross-linking (XL-MS) or hydrogen–deuterium exchange (HDX-MS), which are becoming increasingly informative (Britt, Cragnolini, and Thalassinos, 2021).

On the computational side, molecular dynamics (MD) and Monte Carlo (MC) simulations are commonly used to investigate the conformations and/or dynamics of proteins, often in combination with experimental data to either restrain the structure of the protein or reweight a pool of structures generated from the simulation trajectory to obtain a conformational ensemble that complies with the experimental readout (Lindorff-Larsen et al., 2005; Hummer and Köfinger, 2015; Childers and Daggett, 2018; Orioli et al., 2020). Recent advances in force field (FF) development combined with enhanced sampling techniques now enables a more realistic exploration of protein dynamics and flexibility even in the absence of experimental data (Yang et al., 2019; Abriata and Dal Peraro, 2021). Besides the advances achieved in developing FFs that excel on IDPs (e.g., CHARMM36IDPSFF, Amber ffIDPs, and ffIDPSFF) (Huang and MacKerell, 2018; Zapletal et al., 2020; Mu et al., 2021), the major focus nowadays is on those achieving a balanced sampling on both folded and disordered proteins [such as CHARMM36m (Huang et al., 2017), Amber ff19SB (Tian et al., 2020), and DES-Amber (Piana et al., 2020)]. The main advantage of these simulations is their capability to account for context-dependency (e.g., temperature, ionic strength, PTMs, and a partner). However, their disadvantage is their computational cost, which prohibits proteome-wide/large-scale systematic analyses. To this end, various fast and computationally inexpensive sequence-based predictors have been developed, with many focusing on estimating intrinsic disorder. Disorder predictors can be cataloged into three main categories given their underlying prediction model: (1) *ab initio* methods like IUPred (Dosztanyi et al., 2005), which are based on the protein’s physicochemical properties; (2) machine learning algorithms trained on experimental annotations like Disomine (Orlando et al., 2022), Disopred (Ward et al., 2004), DisEMBL (Linding et al., 2003), and SPOT-DISORDER2 (Hanson et al., 2019); and (3) the meta-predictors that combine several individual predictors, such as PONDR-FIT (Xue et al., 2010), ESpritz (Walsh et al., 2012), DISOPRED3 (Jones and Cozzetto, 2015), MFDp2 (Mizianty, Uversky, and Kurgan, 2014), and others. Usually, most of these predictors of protein disorder focus on labeling regions of missing electron density as regions of disorder using X-ray crystallography or NMR data, categorizing each residue in only one of two classes, ignoring potentially useful conformational states of the protein. However, there are new predictors that address those kinds of different behaviors, like IUPred2A (Meszaros et al., 2018), ODINPred (Dass, Mulder, and Nielsen, 2020), and DispHred (Santos, Iglesias, and Pintado, et al., 2020), assigning a degree of disorder to each amino acid and other predicted features of the protein indicating the amount or degree of disorder, like NetSurfP-2.0 (Klausen et al., 2019) that outputs solvent accessibility, secondary structure, structural disorder, and backbone dihedral angles for each residue of the input sequences. The intrinsically semi-disordered state has also been studied, with predictors able to identify such behavior often associated with induced folders and aggregation-prone regions (Zhang et al., 2013, Zhang et al., 2017; Katuwawala et al., 2019). In addition, other sequence-based predictors provide useful information, such as backbone dynamics (DynaMine) (Cilia et al., 2013, 2014), fuzziness (FuzPred) (Horvath et al., 2020; Miskei et al., 2020), secondary structure [PSIPRED4 (Jones, 1999), SPOT-1D (Singh et al., 2021)], solvent accessibility [SABLE (Adamczak, Porollo, and Meller, 2004), ACCpro (Magnan and Baldi, 2014), SPOT-1D (Singh et al., 2021)], solubility/aggregation propensity [TANGO (Fernandez-Escamilla et al., 2004), AGMATA (Orlando et al., 2020), PASTA2 (Walsh et al., 2014), CamSol (Sormanni, Aprile, and Vendruscolo, 2015)], liquid-liquid phase separation propensity [catGRANULE (Bolognesi et al., 2016), PScore (Vernon et al., 2018), PSPer (Orlando et al., 2019, p.), Droppler (Raimondi et al., 2021)], and other biophysical features of proteins. As most of these prediction tools only take the sequence as input, with sometimes a few specificities or sensitivity parameters, they remain largely context-independent and cannot take factors such as pH, temperature, or PTMs into account. The exception is a few specific cases, such as (i) oxidation-dependent disorder prediction by IUPred2A (Mészáros et al., 2018, p. 2); (ii) pH-dependent solubility prediction for IDPs by SolupHred (Santos et al., 2020a; 2020b; Pintado et al., 2021); (iii) prediction of molecular recognition features/elements (MoRFs/MoREs) that are interacting regions of IDPs undergoing an increase in the secondary structure propensity upon binding (e.g., α-MoRF-PredII predictors (Oldfield et al., 2005; Cheng et al., 2007), MORFchibi (Malhis, Jacobson and Gsponer, 2016), SPOT-MoRF(Hanson et al., 2020), and fMoRFpred (Yan et al., 2016)); and (iv) experimental condition (pH, temperature, ionic strength, crowding agent, and protein concentration)-dependent prediction of liquid-liquid phase separation by Doppler (Raimondi et al., 2021).

Another significant influence on protein behavior is post-translational modifications (PTMs), which regulate the function, activity, and stability of proteins. Several studies have shown the association of PTMs with various diseases, such as cancer, Alzheimer’s, and diabetes (McLaughlin et al., 2016; Song and Luo, 2019; Bai et al., 2021). PTMs alter the biophysical, thermodynamic, and kinetic properties of proteins, leading to a more diverse conformational landscape than dictated by the arrangement of 20 amino acids (Shental-Bechor and Levy, 2008). Therefore, a complete comprehension of a folded protein monomer is useful but insufficient to understand the functioning of a protein in a biological environment. The structural preferences of PTMs are divided into two categories: well-defined secondary structures (N-linked glycosylation, acetylation) and intrinsically disordered regions (phosphorylation, methylation). These PTMs can exist simultaneously in different amino acids (methylation, phosphorylation), or in the same amino acid over time (ubiquitination, phosphorylation), depending on the biological context. The impact of PTMs on protein structures can vary diversely, ranging from local conformational stabilization or destabilization of secondary structure elements to transitions between intrinsically disordered and ordered states (Bah and Forman-Kay, 2016).

In the case of IDPs, the disorder-to-order transitions can be considered “a black box of structural biology.” This multifaceted folding/unfolding behavior is widely regulated and modulated by PTMs. The alteration of IDPs’ conformational space, dynamics, functionality, cellular expression, and localization caused by PTMs can also be unfavorable and cause protein pathogenicity. This equivocal relationship between PTMs and IDPs significantly enlarges the complexity of the black box, which is invisible yet an important attribute of protein folding (Bah and Forman-Kay, 2016). Currently, the change in conformational dynamics of a protein when modified by a PTM can be investigated by MD simulations. However, the systematic force-field parameters required for MD simulations are limited to several PTMs (methylation, phosphorylation, glycosylation) and require optimization and validation, which is computationally expensive. It, therefore, remains a black box since the current tools are deficient in terms of exploring PTMs and the conformational behavior of proteins. On the other hand, the stability of folded regions can also be affected by PTMs. Incorporating information about PTMs into our understanding of *in vivo* protein behavior is, therefore, essential.

**FIGURE 1:**
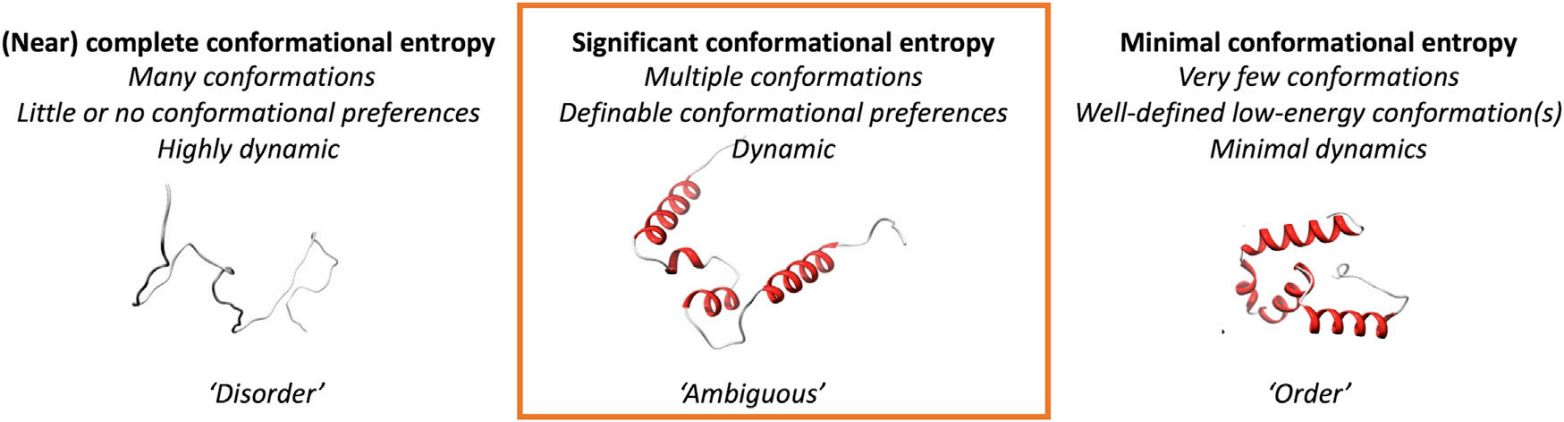
Conceptual definition of the “ambiguous” regions of proteins addressed in this study.

We here explore a class of protein regions that are more likely to adopt multiple different conformations and show ambiguous behavior; they can neither be strictly classified as traditional “order,” nor as the oppositely defined “disorder” (Figure 1). We focus on three different scenarios of conformational ambiguity: (i) regions that undergo “order-to-disorder” transitions, where a protein (region) that is disordered folds when encountering a binding partner, (ii) regions of folded proteins that can change their conformation, and (iii) regions that have ambiguous behavior in solution based on NMR chemical shift information. Such inherent ambiguous behavior could be relevant for conformational changes in the protein, for example, upon oligomerization, interacting with another molecule or the cell membrane, or when being post-translationally modified. These changes should happen within the context of biologically reasonable environments and protein modifications, for example, in disorder-or-order inducing agents such as TFE, or denaturing agents like urea. We here show, based on two different definitions and their joint one, that ambiguous regions are difficult to define but that combinations of datasets from different sources might help to unravel this complex protein behavior.

## 2 Materials and methods

### 2.1 Datasets

#### 2.1.1 DisProt “folding-upon-binding” dataset with CoDNaS dataset (disprot_codnas_set)

DisProt is a large database of manually curated intrinsically disordered protein (IDP) regions (IDRs) (Hatos et al., 2020). Besides the structural state and the function of the region, if available, interaction partners and potential structural transitions (e.g., displaying folding-upon-binding) are also annotated for DisProt entries. For the present study, we downloaded a custom set of human proteins with manually curated disorder-to-order structural transitions, resulting in 138 different proteins with at least one IDR that undergoes ordering. The residues that are classified as undergoing structural ordering were labeled as ambiguous (*N* = 9,792 residues) and the residues in the IDR flanking regions that are not proven to undergo structural ordering were labeled as disordered (*N* = 4,232 residues).

CoDNaS (Monzon et al., 2016) stores proteins with multiple X-ray and NMR structures solved under different experimental conditions. The difference between these conformations of “snapshots” varies over a wide range, with rigid globular structures being on one side of the spectrum and disordered structures on the other side. To assemble a set of rigid proteins, we downloaded structural clusters by applying the threshold of a maximum RMSD value of 2Å for each pair of structures available for the same protein region. This way, we obtained a reliable set of 207 human proteins entailing 11,947 residues in ordered segments.

These two datasets were combined into a single dataset, which, therefore, contains highly reliable definitions for ordered residues (O) for which little or no conformational change has been observed in experimental protein structures (from CoDNaS) as well as disorder (D) (from DisProt) and ambiguous behavior folding-upon-binding residues, with a local change in environment (the binding partner) triggering a conformational transition or rearrangement (T) (from DisProt).

#### 2.1.2 MFIB dataset (mfib_set)

MFIB (Fichó et al., 2017) is a database of mutually folded IDPs/IDRs that synergistically fold upon binding, while as monomers, the protein chains are unstructured. A subset of MFIB was manually selected to reduce the redundancy in terms of a sequence-structure relationship. Additional overlap with other datasets has also been filtered out; in total, five protein chains that were part of the DisProt set have been eliminated. The final set of cases includes 17 chains from homo- and 23 chains from heterocomplexes forming various types of folds (including histone-like folds; basic helix-loop-helix; Phe-, Leu-, and Ala-zippers; and ribbon-helix-helix folds), with 1–3 examples selected from each fold category. The complete dataset is available at https://bitbucket.org/bio2byte/protein_ambiguity/.

#### 2.1.3 Metamorphic and fold-switching proteins dataset (foldswitch_set)

The fold switchers dataset is a manually curated list of pairs of experimentally solved structures for the same protein that shows a different topology in some parts of the sequence. This dataset provides experimental proof of residues that can switch from one secondary structure element type to another one (e.g., a residue that in one of the PDB structures is in an α-helix and in the other one is in a β-strand). The original fold switchers list consisted of 94 protein pairs (PDB entries), but we filtered it to keep only the protein sequences that shared the same sequence, as small sequence variations could have an impact on the protein topology and would, therefore, affect our study. A total of 29 structure pairs remained, totaling 8,047 residues. This dataset is available at https://bitbucket.org/bio2byte/protein_ambiguity/ as supplementary material.

The residues were labeled using the DSSP secondary structure annotations (Kabsch and Sander, 1983) extracted from the PDBe API (Mir et al., 2018) for each of the structures in the pair. Residues that stayed in either helix or sheet conformations were labeled as the same (S), while residues that switched from any secondary structure type to another one were labeled as converted (C). We did not use the residues that stayed in the coil for this analysis to avoid including likely disordered regions in either of the two aforementioned categories. A total of 3,751 and 1,341 residues were labeled as S and C, respectively.

#### 2.1.4 Combined dataset (combined_set)

A new dataset merging the disprot_codnas_set and foldswitch_set was generated by combining some of the categories of the previous ones (combined_set). The ordered (O) and same (S) categories from the disprot_codnas_set and foldswitch_set were merged as they were comparably defined. In both cases, the residues that fall into these categories are amino acids that have proved rigid/conformationally stable in several experimental assays. Similarly, the ambiguous folding-upon-binding residues (T) from DisProt and the fold-switching residues (C) also share a particular biophysical behavior, as in both categories the residues undergo conformational rearrangement. The goal is to assess whether this dataset exhibits similar features with respect to the disprot_codnas_set and foldswitch_set or whether it captures different biophysical characteristics. The disordered category (D) remains as defined in the disprot_codnas_set. The total number of residues in this set is 15,698, 10,750, and 4,232 for ordered (O + S), ambiguous (T + C), and disordered (D), respectively.

#### 2.1.5 Post-translational modification dataset (ptm_set)

PTM information was obtained from four different resources: Scop3P (Ramasamy et al., 2020), UniProtKB/Swiss-Prot (The UniProt Consortium, 2021), dbPTM (Huang et al., 2019), and PhosphoSitePlus (PSP) (Hornbeck et al., 2015). Scop3P annotates protein phosphorylation sites by re-processing large-scale public proteomics datasets. dbPTM integrates experimentally validated PTM sites from Swiss-Prot, PhosphoELM, and O-GLYCBASE. UniProtKB includes PTM information that is directly curated from scientific literature and propagates the information to homologues. PSP contains manually curated PTM information obtained from the literature. We downloaded PTM information from all the above-mentioned resources (April 2022). All the obtained PTM sites were checked for correctness in sequence positions with the current UniProtKB/Swiss-Prot human protein sequences. To obtain a reliable set of PTM sites, we only considered sites having at least two different databases of evidence. Multiple sites having more than one PTM type are labeled as “multiple.” The final dataset contains 217,082 PTM sites from 15,420 canonical human proteins. The complete data table is available at https://bitbucket.org/bio2byte/protein_ambiguity/.

#### 2.1.6 Alphafold human proteome dataset (af_set)

AlphaFold 2’s mmCIF files for the human proteome were downloaded on 2 September 2021, from the AlphaFold protein structure database (Tunyasuvunakool et al., 2021). In this section, we will refer to this dataset as “AF_dataset.” According to AF_dataset’s description page (https://alphafold.ebi.ac.uk/download), sequences longer than 2,700 residues were split into multiple files. For simplicity, we removed these sequences and kept only the sequences contained in a single file. Then, we extracted the protein ID, sequence, pLDDT, and secondary structure and simplified them to alpha_helix, beta_strand, and all remaining conformations were labeled as the coil.

We also downloaded all human Swiss-Prot entries contained in Uniref90 (Suzek et al., 2007) on 2 September 2021 from UniProt (The UniProt Consortium, 2021). In this section, we will refer to this dataset as “uniref_dataset.” From this set, we discarded all proteins shorter than 20 amino acids since some of our predictive tools have this minimum length requirement. Then, we found the sequence intersection between AF_dataset and uniref_dataset and verified that the sequence in both sets was correctly aligned, which resulted in the “selected_human_dataset”.

With these sequences, we computed sequence-based predictions with the b2btools predictors, comprising DisoMine (disorder) (Orlando et al., 2022), DynaMine [backbone (Cilia et al., 2013) and side-chain dynamics, conformational propensities (Raimondi et al., 2017)], EFoldMine (early folding propensity) (Raimondi et al., 2017) using a recently developed PyPI package currently in open beta (https://pypi.org/project/b2bTools/3.0.0b16/). We then merged our predictions with the mLDDT and secondary structure predictions that we extracted from the AF_dataset into our selected_human_dataset. Finally, our selected_human_dataset was saved into a NumPy file for later processing and can be found at https://bitbucket.org/bio2byte/protein_ambiguity/.

#### 2.1.7 Deleterious mutant datasets

Even though mutation is a random process, it frequently occurs at highly conserved hotspots of the protein, which represent regions of structural and functional importance (Chang et al., 2018). To explore the definition of ambiguous regions, we downloaded publicly available deleterious somatic mutations from the catalog of somatic mutations in cancer (COSMIC version92_1,121) (Forbes et al., 2008) and Cancer Genome Interpreter (Tamborero et al., 2018) and germline deleterious and benign mutations from ClinVar (Landrum et al., 2018) and UniProtKB/Swiss-Prot (The UniProt Consortium, 2021), respectively. The COSMIC database contains more than 13 million mutations associated with various cancer types. UniProtKB/Swiss-Prot contains variant annotation from literature reports and ClinVar reports on the relationships among human variations and phenotypes, with supporting experimental evidence from the literature.

Two different analyses were performed. For the first one, 9,295 missense mutations were selected and mapped on 1,115 canonical UniProt ids with at least one deleterious and one benign mutation, resulting in 4,690 deleterious and 4,605 benign mutations. The second analysis focused on comparing somatic and germline deleterious missense mutations shared among 173 canonical isoforms, resulting in 2,145 somatic and 1,020 germline mutations. The datasets are available under the names “canonical_mut” and “germline_somatic_deleterious” at https://bitbucket.org/bio2byte/protein_ambiguity/.

### 2.2 Predictions

#### 2.2.1 Feature generation from sequence

For all protein sequences in the datasets, seven biophysical features were predicted at the residue level using the following methods: backbone dynamics (DynaMine) (Cilia et al., 2013), side-chain dynamics (Raimondi et al., 2017), conformational propensities (helix, sheet, and coil) (Raimondi et al., 2017), early folding propensity (Raimondi et al., 2017), and disorder (DisoMine) (Orlando et al., 2022).

#### 2.2.2 Random forest predictor for folding-upon-binding regions of proteins

The disprot_set describes protein regions that are initially disordered but fold upon binding, with a local change in environment (the binding partner) triggering a conformational rearrangement, while the codnas_set describes residues for which little or no conformational change has been observed in experimental protein structures. The disprot_set was used to define ambiguous/transitioning residues (T) as well as disordered residues (D) and whilst ordered residues (O) were defined from the codnas_set. We used a combination of these datasets (disprot_codnas_set) to train a random forest (RF) predictor, termed folding_upon_binding_RF, with the main aim of creating an interpretable predictor, not necessarily a predictor with the best possible performance. The classification model was trained using seven predicted biophysical features at the residue level (see the previous section). No amino acid codes were used in the training, with all the features computed using a local version of b2BTools from the single input sequences (Kagami et al., 2021). The previously defined residue categories (O, T, and D) were used as labels for the RF training. We used scikit-learn (Pedregosa et al., 2011) version 1.0.2 to generate all the models. The available information for the 25,588 residues was split into 90% and 10% between the training and test sets, respectively. For the training, a 3-fold cross-validation was performed to select the best hyperparameters (n_estimators = 75, max_depth = 15, min_samples_split = 5, min_samples_leaf = 1, and bootstrap = False). The RF model is trained using those hyperparameters and finally tested on the remaining 10% of the data (test set), from which our model is completely agnostic.

#### 2.2.3 Combined random forest

The combined_set was generated by merging the ordered (O) and same (S) categories, and the transition (T) and convert (C) categories from the disprot_codnas_dataset and the foldswitch_set, respectively (for details, see c. f. *Datasets*). Again, the same biophysical predictions were used at the residue level as features for an RF classifier (combined_RF). The data was split 70% to 30% into train and test sets, respectively. The best hyper-parameters were retrieved using a 3-fold cross-validation (n_estimators = 25, max_depth = 15, min_samples_split = 5, min_samples_leaf = 5, and bootstrap = True) and the model was further validated by testing it on the test set that contains 30% of the original data.

#### 2.2.4 Interpretation of random forest models

The RF models were interpreted using a surrogate model trained over the predictions for each of the models. To generate these models, we used the Weka (Eibe et al., 2016) implementation of the Ripper algorithm (Cohen, 1995) (Repeated Incremental Pruning to Produce Error Reduction) that works as a rule-based classification algorithm and supports multi-classification tasks. As a result, we obtained a limited set of rules that summarize the key information on the RF models to classify the residues into different categories. The surrogate models simplify the complexity of the original RF, making them easier to interpret, as the decision trees derived from the raw RF models are often too big and diverse to interpret without any further actions.

## 3 Results

In the first section, we describe the RF predictors of “ambiguous residues.” We did not develop these predictors for optimal performance, but instead for interpretability in relation to the “biophysical” input features. Comparing the predictors, which are each trained on different classifications of ambiguity, enables us to detect whether they seem to recognize the same features (or not), with the aim of identifying whether the different ambiguity definitions (order/disorder transitions or residues that can change conformation in metamorphic/fold-switching proteins) seem to have the same origin. To further contextualize the input features and the classifications, we also describe the relationship of the ambiguous residues to the AlphaFold2 output, as well as information about post-translational modifications and deleterious amino acid variants.

### 3.1 Random forest model interpretation

**FIGURE 2:**
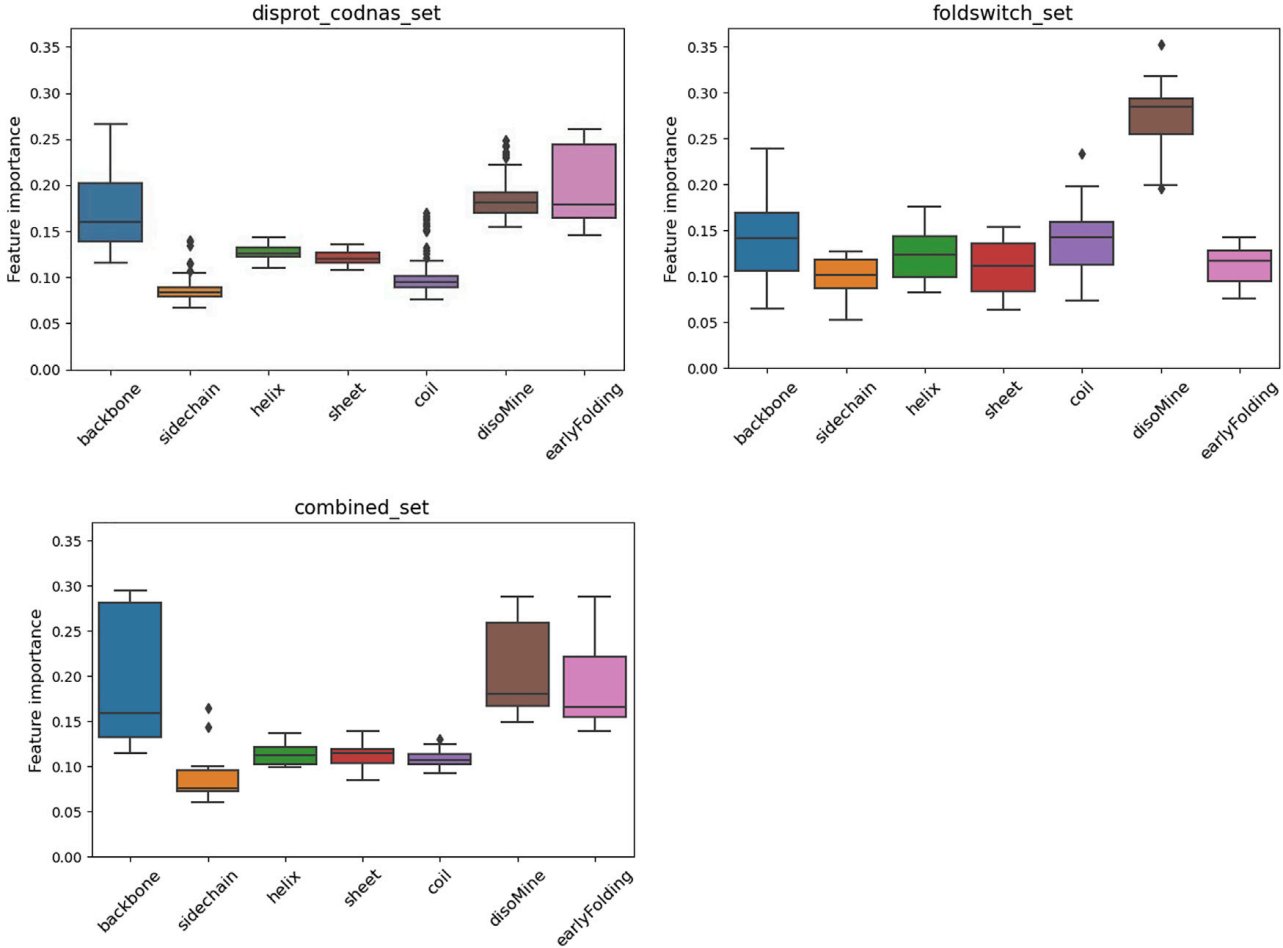
Feature importance variation for the RF classifier for the disprot_codnas_set (top left), the foldswitch_set (top right), and the combined_set (bottom left).

The F1 scores for the folding_upon_binding_RF model to recognize folding-upon-binding regions of proteins based on the combined disprot_codnas_set are lowest for the disorder class (D), where especially the recall is significantly lower (0.67) (Table 1). The performances are overall acceptable and indicate that the model is predictive and captures essential information from the input biophysical features. These features were then ranked by importance (Figure 2), with the early folding (EFoldMine), disorder (DisoMine), and backbone dynamics (DynaMine) being the most relevant. The secondary structure propensities and side-chain dynamics were less relevant for this prediction.

**TABLE 1:** Performances of the trained random forest predictors.

Dataset	Label	Number	Precision	Recall	F1 score
disprot_codnas_set	Order	11,947	0.72	0.84	0.78
	Transition	9,409	0.65	0.6	0.62
	Disorder	4,232	0.72	0.5	0.59
foldswitch_set	Same	3,751	0.79	0.96	0.87
	Convert	1,341	0.72	0.26	0.38
combined_set	Order/Same	15,698	0.72	0.86	0.78
	Transition/convert	10,750	0.62	0.53	0.57
	Disorder	4,232	0.72	0.46	0.56

**FIGURE 3:**
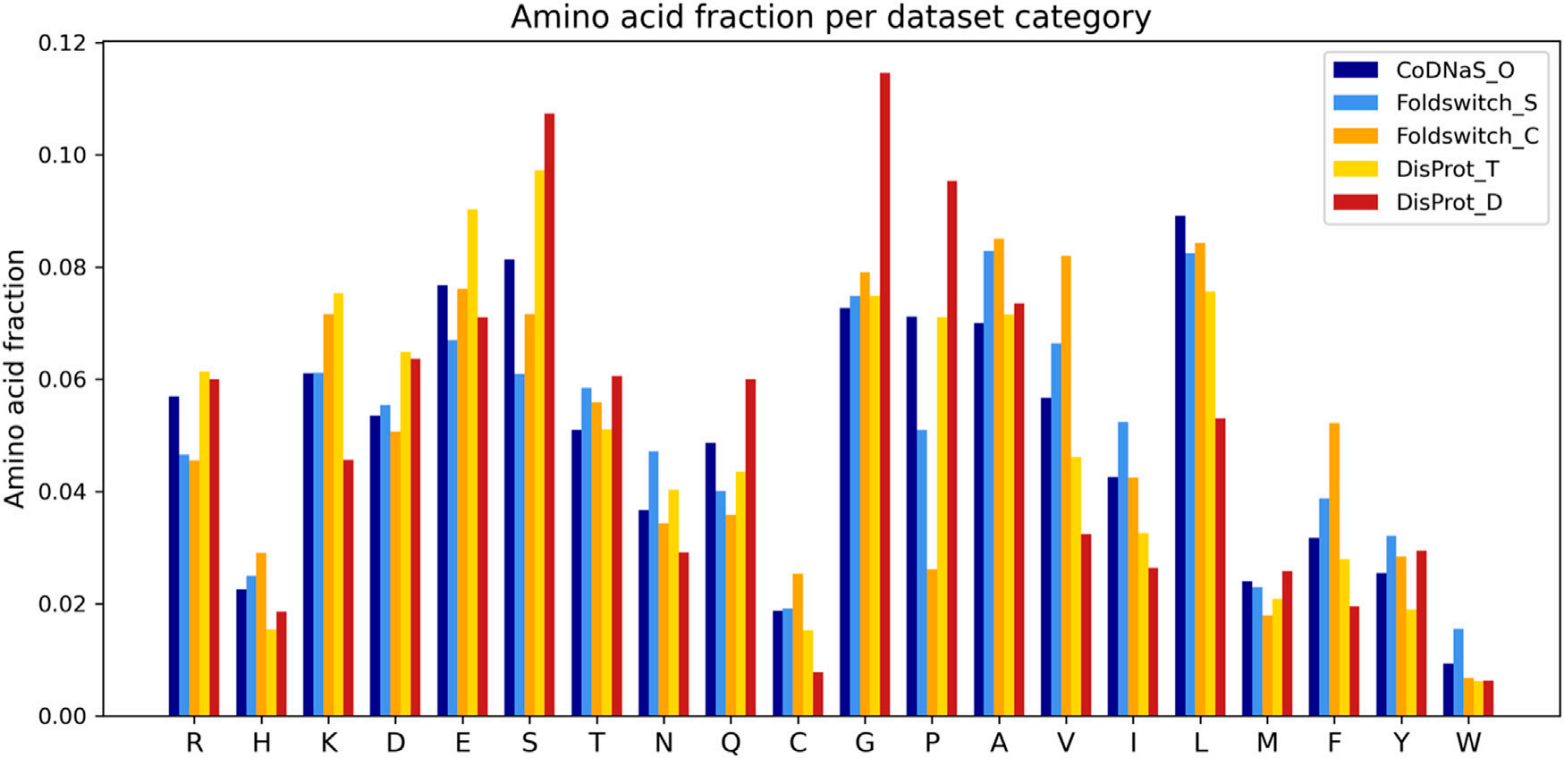
Amino acid fractions observed in the disprot_codnas_set for ordered residues (CoDNaS_O), transition (DisProt_T), and disordered (DisProt_D) and the foldswitch_set for fold-switching residues (Foldswitch_C) and residues that stay in the same fold (Foldswitch_S).

The fold_switching_RF model, based on the foldswitch_set, has a high F1 score for retrieving residues that remain the same when the fold switches (S), but for the residues that convert to secondary structure (C), the F1 prediction performance is very low (0.36) due to very low recall (0.26) (Table 1). This indicates that the biophysical features, which essentially capture local sequence information, are insufficient to detect such residues, or alternatively, that there is little difference between the S and C categories. The amino acid content of fold-switching proteins is similar to those of ordered proteins with a few important differences, including higher valine/phenylalanine and lower proline content for the metamorphic regions (Figure 3). In these regards, this class of proteins is significantly different from intrinsically disordered proteins that have fewer valine and phenylalanine residues but more prolines (Figure 3). In terms of feature importance, the disorder content is the most relevant (Figure 2), indicating that a tendency toward flexibility and/or conformational ambiguity does play a role in distinguishing between the categories, however poor this distinction is.

Finally, the combined_RF model, where the O/S classes and the T/C classes were combined (combined_set), shows overall poorer F1 performances for the O/S classes compared to O and S separately, indicating that the definitions of O and S are likely different, while the T/C class F1 performance is in between the T and C classes, and the D performance drops (Table 1). The feature importance is similar to the one for the disprot_codnas_set (Figure 2). Although there is an imbalance in the absolute numbers of the O compared to S, and T compared to C, classes, the sharp drop in overall performances indicates that the biophysical characteristics required for folding-upon-binding and for fold switching are fundamentally quite different.

The surrogate models generated from each of the RF models provide a perspective on the complexity of the data within. While both the codnas_disprot_set and combined_set surrogate models generate a large number of rules (84 and 89 rules, respectively), the surrogate model trained on the foldswitch_set is much simpler, with just 11 rules, which makes it easier to interpret. We observed that the most disordered residues (DisoMine >= 0.897) are all predicted a transition (ambiguous behavior). Less disordered residues (DisoMine > 0.256) that present a low backbone rigidity (backbone <= 0.724 with DynaMine) are also classified as transition, as are residues with low backbone rigidity (backbone <= 0.754) and a high coil propensity (coil >= 0.505). The rest of the rules are often the combination of three or more biophysical features, with the disorder by DisoMine and backbone dynamics by DynaMine being the most prevalent ones, as already observed in the RF feature importance analysis (Figure 2).

### 3.2 Assessments on independent MFIB dataset

To assess to what extent the RF predictor can recognize the conditional fold of IDPs undergoing mutual folding-upon-binding, we assembled a validation set based on the MFIB database (Fichó et al., 2017) with structural filtering and removal of overlap with other training datasets (for details, see Methods). These proteins are quite different from the classical IDPs, as they are only disordered in the absence of their binding partner or under conditions that prevent their homo-oligomerization. Otherwise, they fold into compact domain-like structures. Thus, we expected to see an enrichment of the predicted ordered and ambiguous conformational class as opposed to the enrichment of the disordered classes.

**FIGURE 4:**
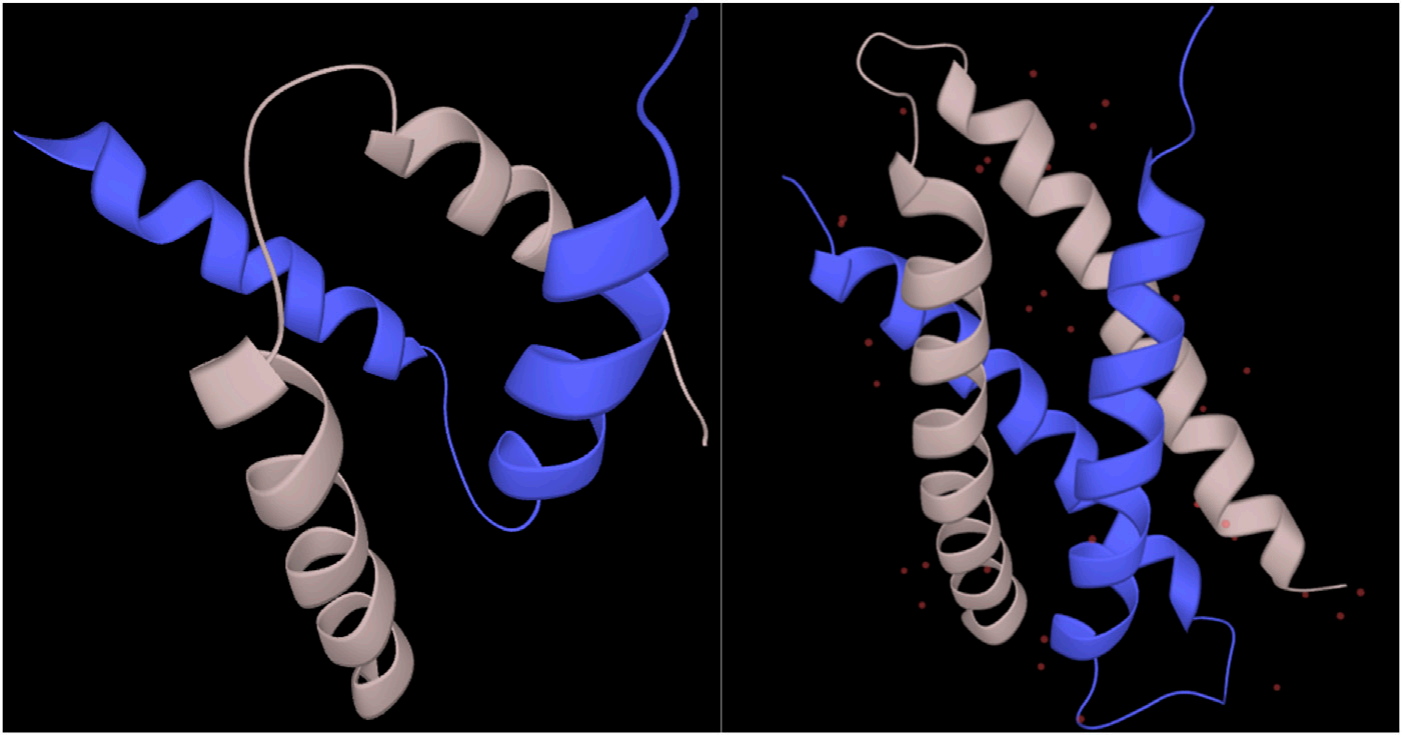
SinR and SH2B2 dimerization domains from MFIB (MF2120029, MF2100004). The SinR (left) dimerization domain (PDB:2YAL) is predicted to have only ambiguous residues, while the SH2B2 (right) dimerization domain (PDB:1Q2H) is predicted to be fully ordered based on the combined_RF model.

For the residues in regions undergoing synergistic folding, the disordered class, without ambiguous folding propensity, was shown to be depleted in the output of the combined_RF predictor (<1%), while the ordered class was predicted to be the most represented (79.6%). The ambiguous class was predicted for 20% of cases, indicating that the folding mechanism of complexes in MFIB, in terms of biophysics, resembles folded domains. This resemblance between folded domains and mutually folded IDPs has already been recognized earlier from the structural and coevolution point of view (Iserte et al., 2020). A significant proportion of ambiguous behavior is still present, however, though fewer than the disorder-to-order transitions of IDPs upon binding or to metamorphic fold-switchers. For individual cases, predictions of regions with ambiguous conformations had significant variation. For example, the SinR dimerization domain of *B. subtilis* (MFIB:MF2120029; PDB:2YAL) is predicted to have ambiguous confirmation with 94% coverage of the domain. On the other hand, the dimerization domain of the human SH2B adapter protein 2 (MFIB:MF2100004; PDB:1Q2H) is predicted to be 100% ordered despite the structural resemblance to the other case (Figure 4). The complete prediction file is available from https://bitbucket.org/bio2byte/protein_ambiguity/.

### 3.3 Relation to AlphaFold2 human proteome models

**FIGURE 5:**
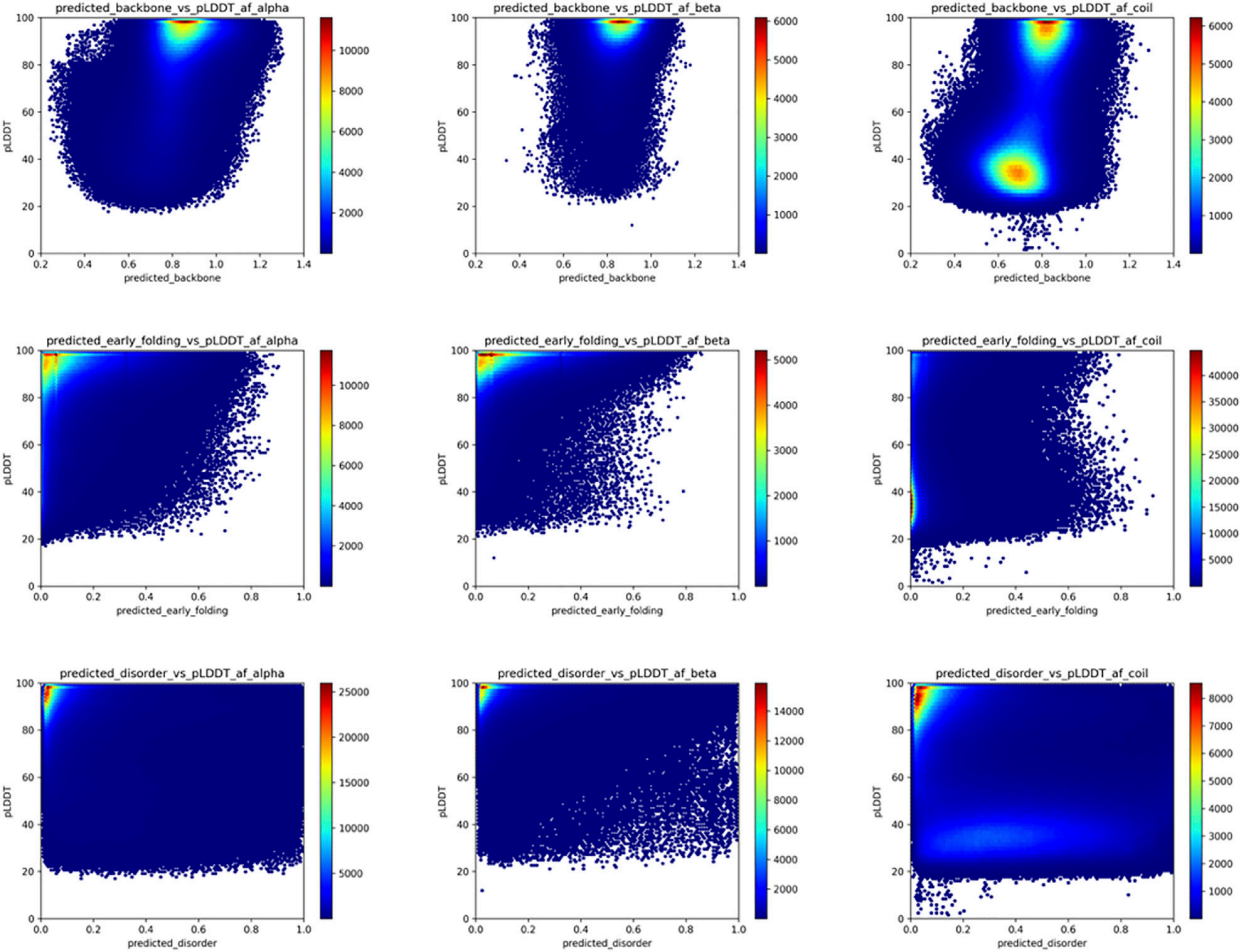
AlphaFold2 pLDDT versus backbone rigidity, early folding and disorder predictions for human proteome residues. Based on the “selected_human_dataset,” we show heat maps of the relation between AlphaFold2 pLDDT value and the backbone rigidity (top), early folding (middle), and disorder (bottom) predictions for residues designed at alpha-helix (left), beta-strand (middle) and coil (right) by DSSP based on the AlphaFold2 models.

AlphaFold2 (Jumper et al., 2021, p. 2, p. 2) can predict single low-energy conformations of proteins with unprecedented accuracy and provides excellent indications of the confidence with which this is done through the per-residue pLDDT values. However, possible conformational ambiguity is not well captured by the AlphaFold2 models (AlphaFold2 fails to predict protein fold switching—Chakravarty—2022—Protein Science—Wiley Online Library, no date), indicating the need to understand how the characteristics of these models relate to conformational ambiguity and dynamics. We, therefore, related the key biophysical predictions of the selected_human_set with the respective pLDDT values of the AlphaFold2 models, subdivided by secondary structure category in the model as determined by DSSP, to understand how these are related, and how this can give insights into the ambiguous residue categories. Figure 5 shows that for the backbone dynamics predictions (first row), the confidently predicted alpha-helix or beta-strand residues, with pLDDT scores close to 100%, have high predicted rigidity (>0.8 DynaMine score); for DynaMine, residues with values above 0.8 are expected to be well folded (Cilia et al., 2014). Residues with a coil classification according to DSSP are either similar to the secondary structure categories (pLDDT confident/backbone rigid), indicating folded residues that do not fall into regular secondary structure categories, or they have low pLDDT confidence and are in the “context-dependent” (DynaMine scores between 0.69 and 0.80), or in the flexible region (<0.69). The pLDDT and DynaMine scores are, therefore, aligned, with high backbone dynamics (lower DynaMine scores) indicating multiple conformations correlating with AlphaFold2 predictions of lower confidence, as it is not able to confidently predict a single low-energy conformation for these residues. The early folding propensity predictions (Figure 5, second row) show that residues with increased early folding propensity are also typically residues predicted with high confidence by AlphaFold2, although AlphaFold2 cannot distinguish between these residues and ones that do not initiate folding pathways, as already indicated by other studies (Outeiral, Nissley, and Deane, 2022). Finally, for disorder predictions (Figure 5, third row), regions with high pLDDT are enriched with residues predicted to have disorder scores of 0 (no disorder), whereas residues predicted to be a coil by AlphaFold2 feature a low pLDDT region that has a wide dispersion of datapoints covering a range of disorder propensity values. Similar to backbone dynamics, this indicates residues that might have ambiguous conformational behavior.

**FIGURE 6:**
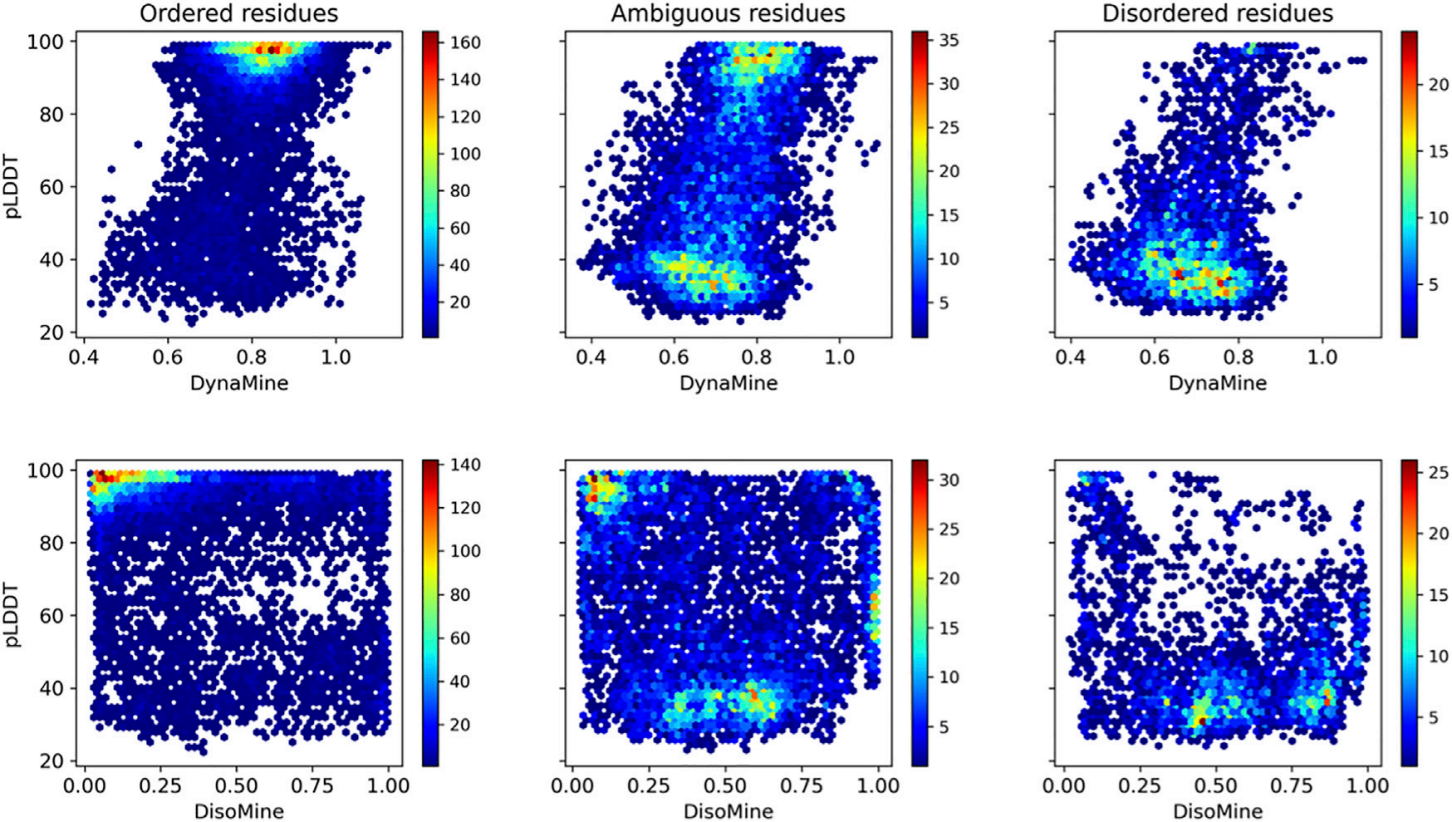
Relation between pLDDT score and backbone dynamics (top) and disorder (bottom) for the O/T/D classes from the disprot_codnas_set.

When subdividing these plots in relation to our datasets that indicate ambiguous residues (Figure 6), these trends are more obvious. The ordered residues cluster at high pLDDT values (>80%) and high backbone rigidity (>0.8), the disordered residues at very low pLDDT values (<40%), and high backbone dynamics (<0.8). The ambiguous residues fall in between these categories, with many lower confidence pLDDT values between 80% and 40%, and backbone dynamics between 0.70–0.80, as well as significant overlap with the ordered and disordered categories. The disorder values confirm this trend, with few ordered residues predicted as having high disorder scores and most disordered residues correctly predicted with high disorder scores. The ambiguous residues again give an intermediate picture, with more residues having scores intermediate between the typical scores for order and disorder.

**FIGURE 7:**
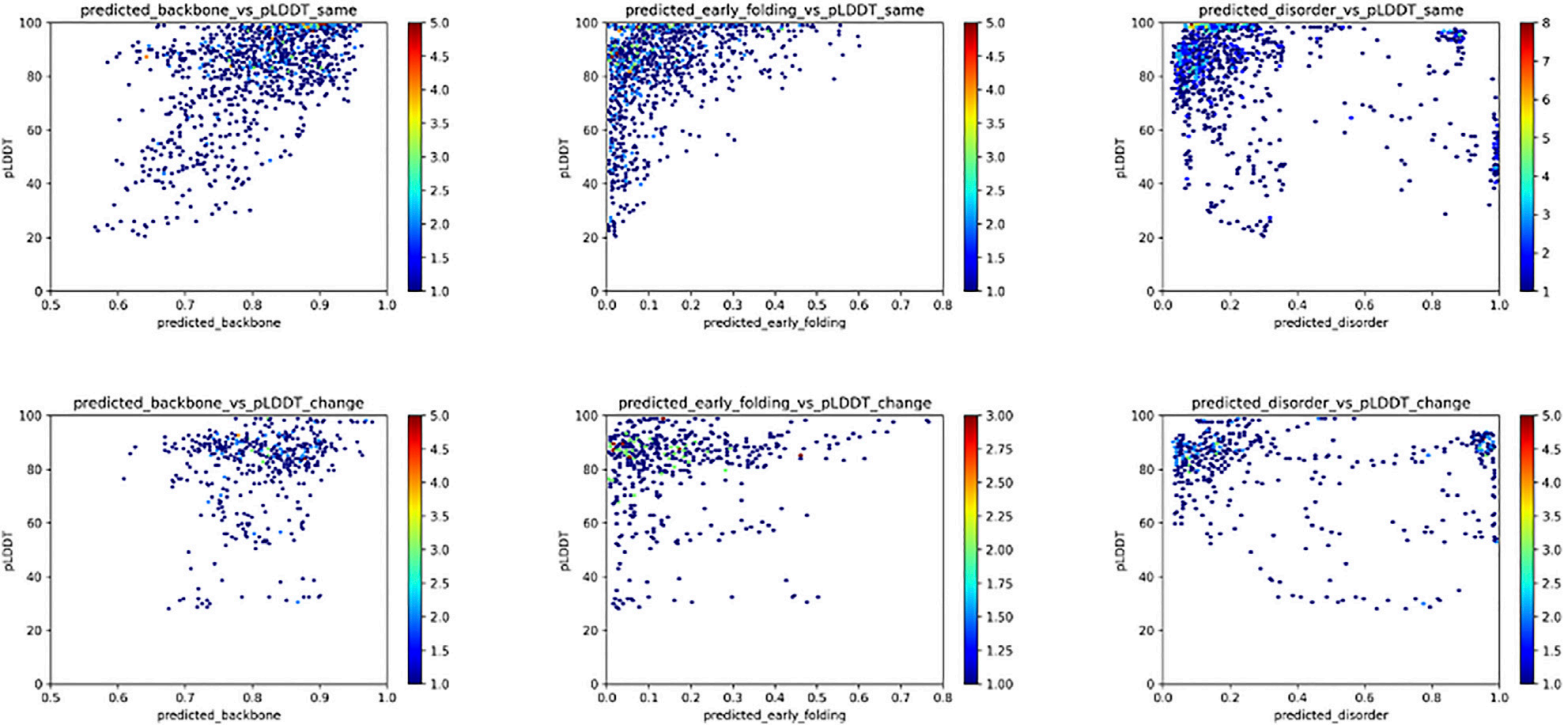
Relation between the pLDDT score and backbone dynamics (left), early folding (middle), disorder (right) for the fold_switch_set same (top row), and convert (bottom row) residues.

For the fold_switch_set only (Figure 7), there are interesting differences, especially the AlphaFold2 pLDDT scores, which tend to be below 90% for the residues that change conformation. The backbone dynamics also contain fewer high values, while more residues are predicted with high disorder.

### 3.4 Relation to post-translational modification data

**FIGURE 8:**
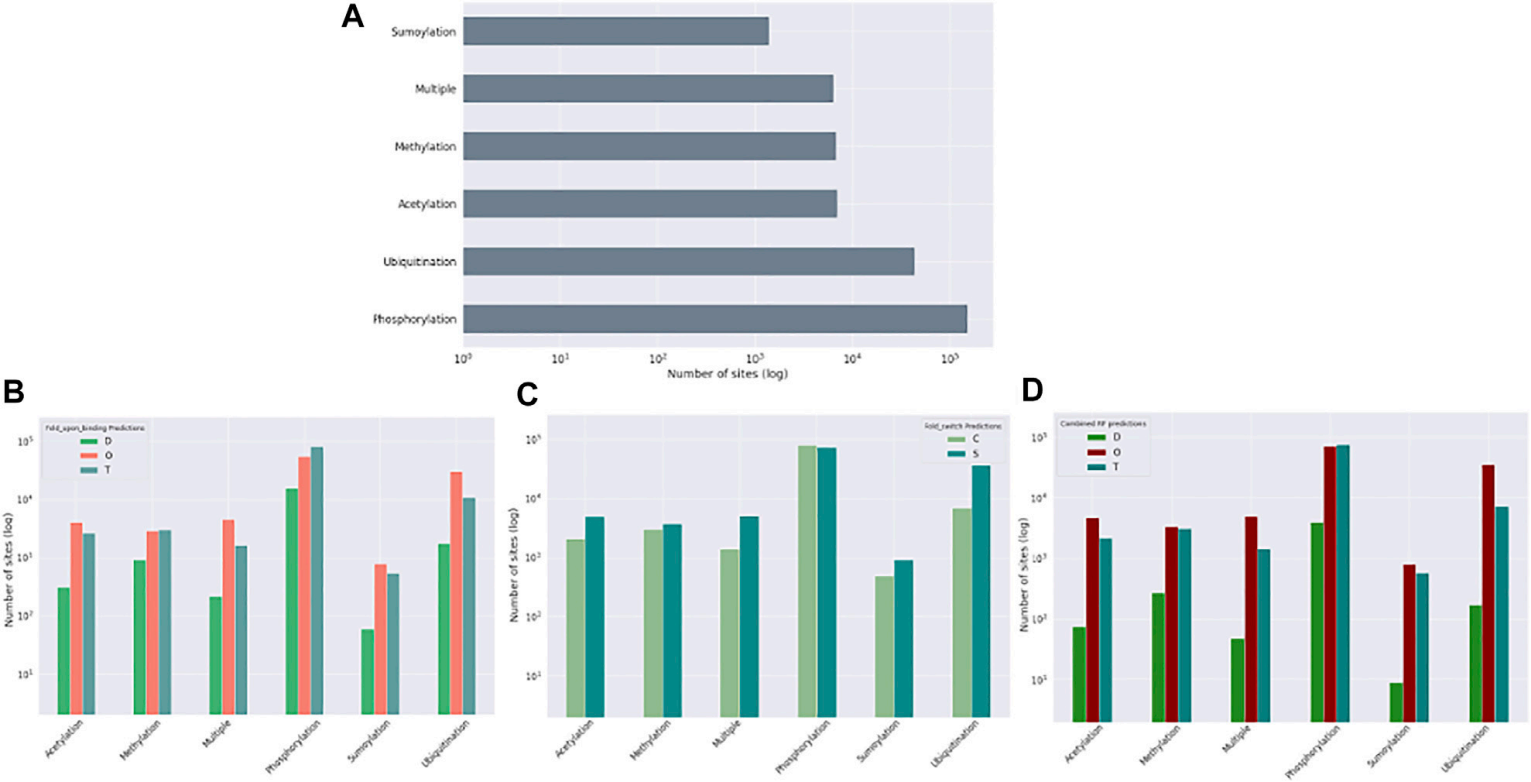
Post-translational modification (PTM) sites from the ptm_set in relation to datasets. The total number of included PTMs **(A)**, subdivided by disordered, ordered, and transition based on disprot_codnas_set **(B)**, by ordered and convert based on foldswitch_set **(C)**, and based on the combined order, disorder, and transition classes **(D)**.

Post-translational modifications (PTMs) of amino acid residues are important for regulation and can have a significant impact on protein conformation and function. Based on the ptm_set, which contains information for sumoylation, methylation, acetylation, ubiquitination, and phosphorylation, or a combination of these (Figure 8, log scale), we subdivided the observed PTMs by the different datasets. For the disprot_codnas_set, the majority of PTMs are observed in the order and transition classes, with phosphorylation overrepresented in residues with transition properties, and with ubiquitination and sumoylation underrepresented (Figure 8B). In the foldswitch_set, residues that remain in the same secondary structure state (S) have again increased ubiquitination and sumoylation compared to residues that convert (C), with a slight increase in acetylation and especially multiple modifications, indicating a possible role in fold-switching processes or more availability of these residues to be modified by smaller PTMs. The trends for the combined_set are very similar to the disprot_codnas_set, which constitutes the bulk of the data.

### 3.5 Relation to deleterious amino acid variants

**FIGURE 9:**
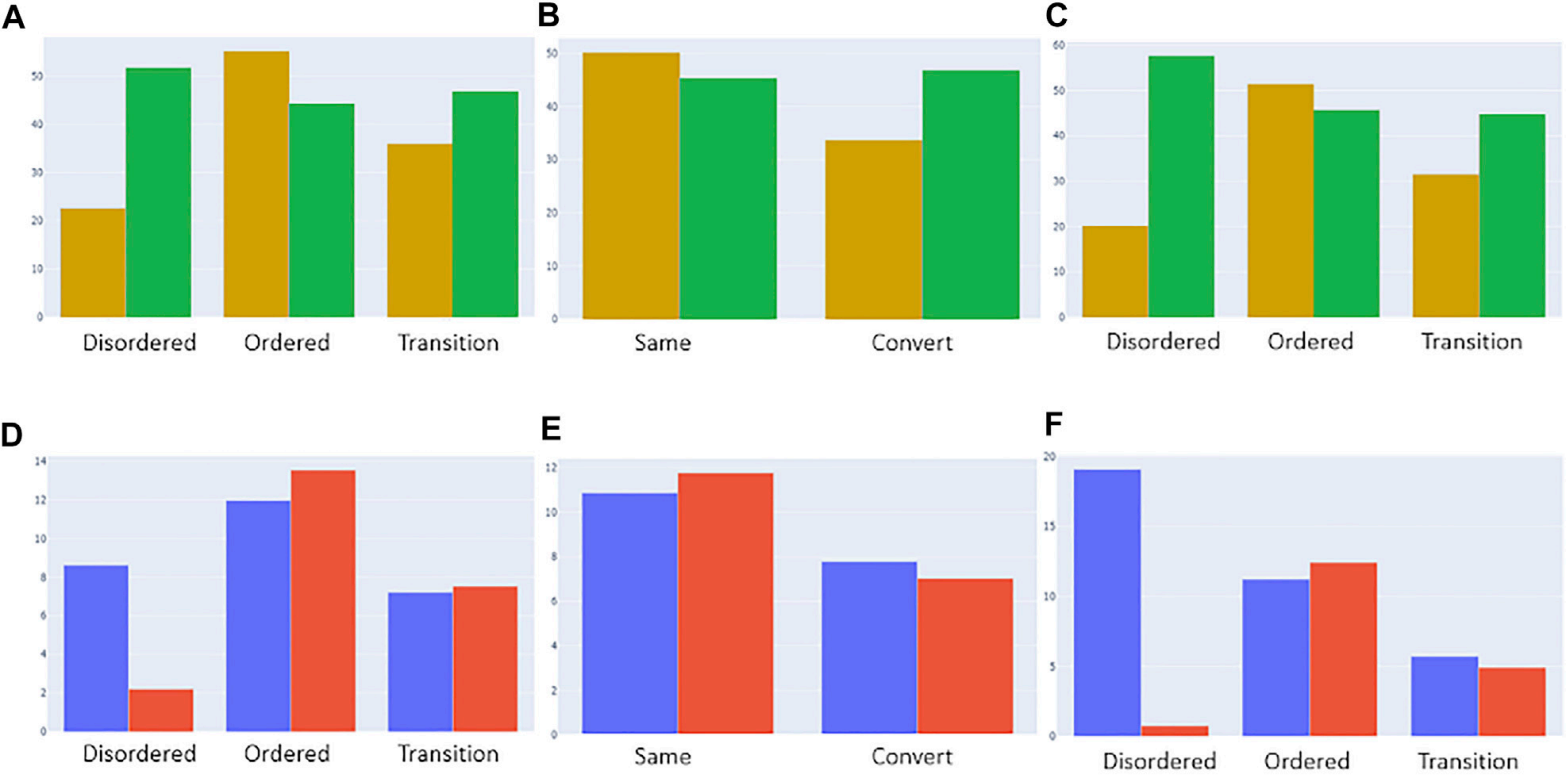
Categorization of deleterious (yellow) and benign (green) mutations from the “canonical_mut” dataset classified as disordered, ordered, and ambiguous based on models 1 [**(A)**, top left], 2 [**(B)**, top middle], and 3 [**(C)**, top right], respectively. The distributions were normalized by the total number of assigned ordered, disordered and ambiguous residues in the dataset. Categorization of germline (blue) and somatic (red) mutations from the “germline_somatic_deleterious” dataset classified as disordered, ordered, and ambiguous based on models 1 [**(D)**, bottom left], 2 [**(E)**, bottom middle], and 3 [**(F)**, bottom right], respectively. The distributions were normalized by the total number of assigned ordered, disordered and ambiguous residues in the dataset and the number of somatic/germline ratios for better comparison.

We also investigated whether residues in ambiguous regions, again given their likely role in conformational rearrangements and allostery, are more likely to contain deleterious or benign mutations, as classified in the canonical_mut dataset. Figure 9A shows that for the disprot_codnas_set (RF model 1), the ordered residues contain, as expected, relatively more deleterious mutations. Although the ambiguous residues contain more benign mutations, they still contain a high proportion of deleterious mutations, especially compared to the ratio observed for disordered residues. This situation is similar to somatic versus germline cancer mutations (Figure 9D). For the foldswitch_set (RF model 2), the ambiguous metamorphic residues contain a higher amount of deleterious mutations than the residues that retain their secondary structure (Figure 9B), whereas there is no difference between somatic versus germline mutations (Figure 9E). For the combined RF model 3, the trends are very similar to RF model 1 (Figure 9C–F).

## 4 Discussion

In this exploratory analysis, we use two datasets that try to capture amino acid residues in proteins that display different “ambiguous” behaviors either by folding-upon-binding (disprot_codnas_set) or by changing secondary structure in metamorphic proteins (foldswitch_set). This definition of “ambiguous” residues is highly relevant given the ready availability of predicted AlphaFold2 protein structure models with qualities comparable to experimentally derived structures. Given the dynamic nature of proteins, and their capacity to change conformation and transmit signals through allostery (Tompa, 2014, 2016), annotations of the AlphaFold2 models indicate where such conformational changes are more likely to happen, which will help in interpreting such models. Our results indicate that AlphaFold2, based on the per-residue pLDDT prediction confidence values, captures ordered and disordered residues very well, and while for ambiguous regions intermediate pLDDT values are observed, many of these ambiguous residues fall into the “traditional” ordered or disordered regions (Figure 6). The RF models we created and their interpretation show that sequence-predicted disorder is the most important factor predicting fold switching residues (from order to order), as well as folding-upon-binding (from disorder to order), with backbone dynamics and early folding also important for the last category. Specific amino acids are also a likely factor, such as valine and phenylalanine for the fold switching residues. Although the recognition by the combined_RF model of the MFIB dataset, which contains dimers that form domain-like structures, is of limited sensitivity (see https://bitbucket.org/bio2byte/protein_ambiguity/), there are indications that ambiguous residues can also be picked up in these cases. This illustrates the complexity of protein behavior in relation to its (local) environment; in this case, and expressed in terms of ambiguous behavior, the local sequence context of the protein is strongly geared toward order, but enough ambiguous residues are present that the individual proteins cannot fold.

Previous AlphaFold2-related studies in this area have given similar indications. AlphaFold2 is a good predictor of intrinsically disordered regions (IDRs) based on the CAID PDB-DisProt dataset (Piovesan, Monzon, and Tosatto, 2022), a study on conditionally folded IDRs (Alderson et al., 2022) showed that many IDRs are in the high (70 ≤ × < 90) or very high (≥90) pLDDT regions, similar to what we report, with enrichment in helical conformations, and with long, extended single α-helix domains not stabilized by tertiary contacts identified. For a subset of IDRs that fold under specific conditions and have been extensively characterized by NMR spectroscopy, the IDRs resemble the conformation of the folded state, even if there is no stable secondary structure observed with only a fractional preference to populate secondary structures from the experimental NMR data. The combination of higher relative solvent accessibility in the AlphaFold2 models, which indicates a lack of overall structure, and high pLDDT scores, which indicate confident structure predictions, does, however, seem to be a good indicator of regions with a tendency for ambiguous behavior (Piovesan, Monzon, and Tosatto, 2022). These results show again that AlphaFold2 is excellent at defining a single low-energy state for a given protein sequence if it exists, but that the context of the protein and possible ambiguous behavior is more difficult to capture. Indeed, in relation to conformational diversity as observed in the PDB from apo-holo pairs of conformers for the same protein (Saldaño et al., 2022), AlphaFold2 predicts the holo form in ∼70% of cases but is unable to capture both states. As the conformational diversity between the apo/holo states increases, its prediction performance also worsens. A similar picture is observed for proteins that can switch folds (AlphaFold2 fails to predict protein fold switching—Chakravarty—2022—Protein Science—Wiley Online Library, no date), with 94% of AlphaFold2 predictions capturing one experimentally determined conformation but not the other, and with moderate-to-high pLDTT scores for 74% of fold-switching residues, similar to our study. Finally, although AlphaFold2 and RoseTTAfold models seem to carry overall foldability information (Liu, Wu, and Chen, 2022), the folding process itself is not well captured (Outeiral, Nissley, and Deane, 2022), if at all.

 Overall, it remains very difficult to capture the dynamic properties of proteins; despite the availability of molecular dynamics simulations of increasing length, limited direct dynamics measurements from NMR and other structural biology approaches, and the observed conformational diversity in the PDB, the complexity of possible protein movements and their likelihood within the *in vivo* environment of proteins, in general, precludes the generation of relevant all-encompassing datasets. The increasing amount of data that indirectly indicates such behavior, from mass spectrometry proteomics (Britt, Cragnolini, and Thalassinos, 2021) as well as from evolutionary and disease mutation sources, will be in this respect invaluable, as already indicated in our limited study. The challenge here lies in interconnecting the various diverse data sources and analyzing the resulting complex information, which is beyond direct human understanding and requires machine learning approaches, preferably interpretable so that concepts and first principles can be derived from them. Furthermore, methodology development in the more traditional sense is also key, for example, improved ensemble representations of proteins and especially IDRs, as already indicated in other studies such as the ones discussed here (Alderson et al., 2022; AlphaFold2 fails to predict protein fold switching—Chakravarty—2022—Protein Science—Wiley Online Library, no date), as well as more accurate sequence-based predictors, with the combination of structure and sequence-based approaches likely giving the most relevant results.

## 5 Conclusion

In our view, it is essential that we move away from the two-state view of proteins (one single well-defined static fold, or complete disorder) to a more nuanced probabilistic view, where the “probability space” of proteins is defined—as the possible states of a protein can adopt. The definition of the different kinds of ambiguity observed in protein behavior, and their interpretation is an important step to help the field move in this direction. Ongoing ELIXIR implementation projects, for example, are also focusing on related topics, highlighting the community's need for this kind of probabilistic interpretation of protein behavior. We hope that the datasets and analyses we assembled here provide additional reference points to further explore and define residues with ambiguous behavior in proteins.

## Data Availability

The datasets presented in this study can be found in online repositories. The names of the repository/repositories and accession number(s) can be found at: https://bitbucket.org/bio2byte/protein_ambiguity/.
